# New form of HPV18 L1 found in native virions contributes to virion stability and infectivity

**DOI:** 10.1128/jvi.00167-26

**Published:** 2026-09-11

**Authors:** Eric J. Ryndock, Jennifer Biryukov, Kumari Asha, Sarah Brendle, Neil D. Christensen, Craig Meyers

**Affiliations:** 1 Department of Cell and Biological Systems, The Pennsylvania State University College of Medicine 12310, Hershey, Pennsylvania, USA; 2 Department of Pathology, The Pennsylvania State University College of Medicine 12310, Hershey, Pennsylvania, USA; Tufts University, Boston, Massachusetts, USA

**Keywords:** human papillomaviruses, HPV18, cervical cancer, viral capsid stability, virus infectivity

## Abstract

Current dogma states that papillomavirus virions consist of only one form of the L1 major capsid protein. Human papillomaviruses (HPVs) have been studied using recombinant particles, thereby bypassing the need to grow the virus in a model of differentiating epithelium. Expression vectors engineered to produce L1 utilize a consensus methionine found in many HPV types. However, HPV18 has two additional in-frame methionine residues located 61 and 26 amino acids upstream of the consensus methionine. These methionine residues are excluded from the L1 expression vector used to create the recombinant virus. On the other hand, HPV produced from organotypic raft culture allows virions to be assembled in a differentiating epithelium in the presence of its native promoters and complete genome. Here, we have utilized this system to show that the wild-type HPV18, produced in a more natural setting, translates a larger form of L1 from the upstream methionine, which is 61 amino acid residues upstream from the consensus methionine, and a smaller form from the consensus methionine, with both sizes assembled in the newly formed virion. Ablation of the upstream methionine residue at position 61 altered the virion capsid conformation, and also decreased virion stability and infectivity.

**IMPORTANCE:**

The present study investigates whether the papillomavirus virions contain more than one isoform of the L1 major capsid protein. By using organotypic raft cultures that mimic the naturally differentiating epithelium, we revealed that wild-type HPV18 expresses and incorporates two distinct L1 isoforms into the virion, including a previously unrecognized longer form translated from an upstream methionine. Our study has further shown that disruption of the upstream methionine impairs virion stability and infectivity, demonstrating that the longer L1 isoform contributes to proper capsid architecture and viral function. These findings offer a new understanding of HPV capsid biology and have significant implications for the future development of HPV vaccines, diagnostics, and antiviral therapies.

## INTRODUCTION

**Fig 1:**
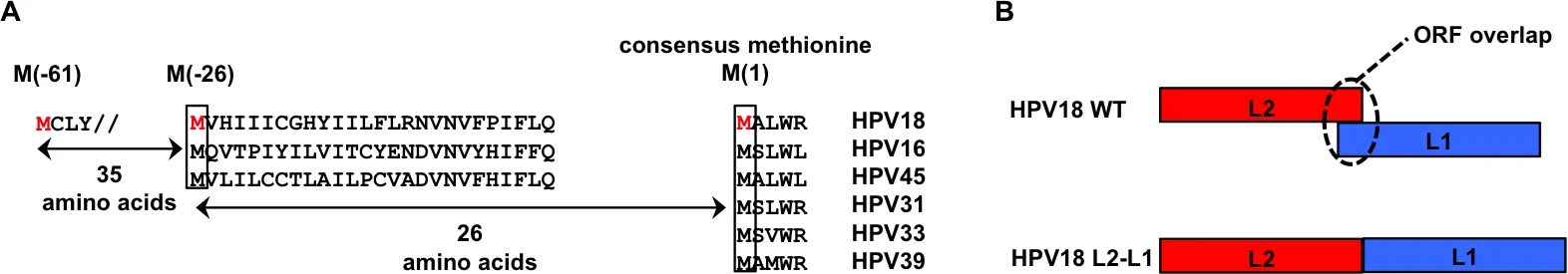
(**A**) Alignment of the N terminus of L1 protein across different HPV types. HPV18 has three notated Met residues: M(−61), M(−26), and the consensus M(1). (**B**) Diagram illustrating the native genetic configuration of overlapping capsid ORFs compared to the engineered HPV18 L2-L1 genome with non-overlapping L1 and L2 ORFs.

Recombinant transfection systems using cells in monolayer have been developed to generate virus-like particles (VLPs), pseudovirions (PsVs), and quasivirions (QVs) in order to study HPV independent of cellular differentiation (1–3). These systems utilize codon-optimized expression vectors with enhanced promoters to produce the capsid proteins L1 and L2. VLPs are self-assembled L1 only or L1 and L2 particles, while PsVs are particles that may contain reporter proteins, such as green fluorescent protein (GFP), along with viral capsid proteins, to be easily tracked in cell entry experiments. QVs, on the other hand, are made using the same capsid gene expression technology as VLPs and PsVs, but their capsids are self-assembled around whole HPV genomes encoding all the viral proteins. The discovery of VLPs has been of great importance, as it has led to the development of a vaccine strategy against HPV infection and cervical cancer (4). For HPV16 L1, the expression vector encoding a translation start site at (nt 5637) is considered the consensus methionine (Met), which can be observed by aligning the N terminus of L1 across different HPV types (Fig. 1A).

The consensus Met was determined to be the most efficient producer of L1 protein in monolayer culture and produced recognizable VLPs (5). In some HPV types, however, the consensus Met is not the first in-frame Met in the L1 open reading frame (ORF) (6). Previous reports show that the expression of HPV16 L1, by itself, from an expression vector using the first in-frame Met residue (nt 5559), 26 amino acids upstream of the consensus Met, produced L1 in monolayer but did not form particles that could resemble virions (7, 8). This has led to all in-frame methionines that exist upstream of the consensus Met, for all HPV types, to be excluded from current HPV L1 expression vectors.

The use of organotypic raft culture, a differentiating epithelial human tissue model, allows HPV to be studied in its more natural environment in the context of its complete genome and native promoters (9–13). This system uses epithelial cell lines stably maintaining the entire HPV genome; therefore, all upstream, in-frame L1 Met residues are present. The HPV18 genome has two additional in-frame Met residues, which are located 61 [M(−61)] and 26 [M(−26)] amino acids upstream of the consensus Met [M(1)] (Fig. 1A). Previously, we have shown that deletion of the region encompassing M(−61) up to M(−26) causes detectable changes in the virion’s capsid conformation, suggesting that this region of HPV18 L1 is important for determining HPV capsid structure (14). In this manuscript, using HPV18 as a model, here, we explored the role of the two upstream in-frame L1 Met residues in the complete viral life cycle and virion stability.

## MATERIALS AND METHODS

### Mutagenesis

pBSHPV18 L2-L1 (14) and pBSHPV18 wild-type DNA (10) were utilized as templates for site-directed mutagenesis using a QuikChange II XL site-directed mutagenesis kit (Stratagene). Methionine (M)-to-alanine (A) substitution mutants, using pBSHPV18 L2-L1 as a template, were created using the complementary oligonucleotides: forward 5′-GCGGCCTAGAAGCTTGCGTGCCTGTATACACG-3′ and reverse 5′-CGTGTATACAGGCACGCAAGCTTCTAGGCCGC-3′ for M(−61)A L2-L1; forward 5′-CTACACAGTATATTGGTATACGCGGTACACATTATTATTTGTGG-3′ and reverse 5′-CCACAAATAATAATGTGTACCGCGTATACCAATATACTGTGTAG-3′ for M(−26)A L2-L1; and forward 5′-CTATTTTTTTGCAGGCGGCTTTGTGGCGGCC-3′ and reverse 5′-GGCCGCCACAAAGCCGCCTGCAAAAAAATAG-3′ for M(1)A L2-L1. Double and triple methionine-to-alanine substitution mutants were created by using the positive clones from the single substitution mutants as a template in combination with the relevant corresponding primers. Methionine-to-alanine substitution mutants using pBSHPV18 wild type as template were also created using the complementary oligonucleotides: forward 5′-CCCTTTAACCTCCTCTTGGGGCGTGCCTGTATACACGGGTCCTG-3’ and reverse-5′ CAGGACCCGTGTATACAGGCACGCCCCAAGAGGAGGTTAAAGGG-3′ for M(−61)A and forward 5′-CCCTTTAACCTCCTCTTGGGACGTGCCTGTATACACGGGTCCTG-3′ and reverse 5′-CAGGACCCGTGTATACAGGCACGTCCCAAGAGGAGGTTAAAGGG-3′ for M(−61)T. Multiple clones containing the correctly mutagenized viral genomes were isolated for each mutation and utilized in further experiments. All mutants were sequenced.

### Keratinocyte culture and electroporation

Primary human foreskin keratinocytes (HFKs) were isolated and grown from newborn circumcision as described previously (12). For electroporation, 30 µg of the wild type or mutated viral DNA was digested with EcoRI, linearizing the viral DNA and separating it from the vector sequence. HFKs were then electroporated with the prepared DNA as described previously (9, 12). Viral DNA was extracted from each stable cell line produced and sequenced to verify the presence of mutations.

### Southern blot hybridization

Total cellular DNA was isolated from cell lines that stably maintained episomal copies of the HPV genome as previously described (12). Briefly, 5 µg of total cellular DNA was digested with EcoRI, linearizing the HPV18 genome. Samples were then separated by 0.8% agarose gel electrophoresis, transferred onto a GeneScreen Plus membrane (New England Nuclear Research Products), followed by hybridization with the whole-genome HPV18-specific probe to detect the HPV18 DNA, as previously described (12, 15).

### Organotypic raft culture-derived virion production

Immortalized HFK lines, stably maintaining episomal HPV18 DNA, were grown in monolayer culture using E-medium in the presence of mitomycin C-treated J2 3T3 feeder cells as described previously (12). Raft tissues were grown for 20 days as previously described (12, 16). At least two different cell lines and two different batches of organotypic rafts were grown and tested in all experiments to discriminate between batch variations. A description of all variants of the virus used in this study can be found in Fig. S1.

### 293TT monolayer QV production

HPV18 QVs were generated in 293TT monolayer cells as previously described (2). Briefly, 293TT cells plated to 90% confluency in T-150 flasks were co-transfected using Lipofectamine 2000 (Invitrogen) with 25 µg each of HPV18 wild-type genome and a bicistronic HPV18 L1/L2 expression vector (17). Cells were split 1:2 at 24 h post-transfection and harvested 48 h post-transfection. Cell pellets were resuspended in a total of 750 µL of phosphate buffer. Cells were lysed by Dounce homogenization as in the HPV isolation step described below. Finally, MgCl_2_ was added into the lysate at a final concentration of 2 mM. Lysates were then incubated overnight at 37°C for maturation of the HPV18 QV particles (18). Unpackaged DNA was digested by incubating preparations in benzonase (350 U) (Sigma) for 1 h at 37°C. After digestion, NaCl was added to the preparation to a final concentration of 1 M. Cell debris was removed by centrifugation for 10 min at 10,500 rpm and 4°C. The supernatant was harvested as the viral preparation and was stored at −80°C.

### Histology

Tissues were fixed in 10% neutral buffered formalin and embedded in paraffin. Sections (4 µm) were cut and stained with hematoxylin and eosin (H&E) as previously described (9). For immunofluorescent (IF) staining, slides were deparaffinized in xylenes 2 × 10 min, followed by washing for 3 × 3 min in 100% ethanol and rehydration in dH_2_0 for 5 min. Antigen retrieval was performed by boiling slides in Tris-EDTA (pH 9.0) for 10 min. After cooling to room temperature, slides were rinsed in 1× TBS-Tween for 5 min. Background sniper blocking agent (Biocare Medical) was utilized to block tissue for 15 min at room temperature. Tissue sections were then incubated in the appropriate antibody diluted in Da Vinci Green antibody diluent (Biocare Medical) at 4°C overnight. Slides were then washed in 1× TBS-Tween 3 × 5 min prior to incubation with the appropriate secondary antibody, diluted 1:200 in 1× TBS-Tween, for 1 h at room temperature. Slides were once again washed 3 × 5 minutes in 1× TBS-Tween and mounted with ProLong Gold Antifade with DAPI (Invitrogen) mounting media. Sections were imaged on a Nikon Eclipse 80i microscope, and images were acquired using a CoolSnap cf2 camera and NIS-Elements 3.10 software. Images were adjusted for brightness and contrast identically.

### HPV isolation

Three rafts were prepared by Dounce homogenization in 500 µL of phosphate buffer (0.05 M sodium phosphate [pH 8.0]) for virus preparations. Homogenizers were then rinsed with 250 µL of phosphate buffer. Non-encapsidated genomes and cellular DNA were removed by adding 1.5 µL (375 U) of benzonase and 1.5 µL of 2 M MgCl_2_ for 1 h at 37°C. Samples were brought to 1 M NaCl by adding 195 µL of 5 M NaCl. Then, the samples were mixed and centrifuged at 4°C for 10 min at 10,500 rpm in a microcentrifuge. The supernatants were stored at −80°C.

### Optiprep purification of virions

Optiprep purification was performed as described previously (3, 16). Briefly, 27%, 33%, and 39% Optiprep gradients were produced by underlayering. Gradients were allowed to diffuse for 1 h at room temperature. Then, approximately 350 µL of benzonase-treated virus preparation was layered on top of the gradient. Tubes were then centrifuged in a SW55 rotor (Beckman) at 234,000 × *g* for 3.5 h at 16°C. After centrifugation, 11 fractions of 500 µL were carefully collected starting from the top of each tube (top = 1 and bottom = 11). To quantify the differences, relative stabilities were calculated and defined as the ratio of total unprotected genomes to total protected genomes (19). Quantitative RT-PCR (RT-qPCR) and qPCR-based DNA titering assays, both described below, were used to determine the viral titer and infectivity, respectively, in the harvested fractions.

### qPCR-based encapsidation assay

To detect endonuclease-resistant genomes in viral preps or Optiprep fractions, only benzonase-treated viral preps were utilized so that all non-encapsidated genomes were digested (16, 20, 21). To release all encapsidated viral genomes, 10 µL of virus prep or Optiprep fraction was added to 2 µL of proteinase K, 10 µL of 10% sodium dodecyl sulfate, and brought up to 200 µL with HIRT buffer. Tubes were rotated at 37°C for 2 h. Immediately, an equal amount of phenol-chloroform-isoamyl alcohol (25:24:1) was added, and the aqueous phase was extracted. An equal amount of chloroform was added, and the aqueous phase was extracted again. DNA was ethanol-precipitated overnight at −20°C. After centrifugation, the DNA pellet was washed with 70% ethanol and resuspended in 20 µL of Tris-EDTA overnight. To detect viral genomes, a SYBR Green PCR kit (Bio-Rad) was utilized. Amplification of the viral target was performed in 0.2 mL, 96-well PCR plates (Bio-Rad) with a total reaction volume of 25 µL. Then, 1 µL of each endonuclease-resistant viral genome prep was analyzed in duplicate for each independent experiment. Amplification of HPV18 genomes was performed using primers complementary to the HPV18 E2 gene: forward 5′-CAGTATTAACCACCAGGTGGTGGTG-3′ and reverse 5′-GTTCAAGACTTGTTTGCTGCATTGTCC-3′. Oligonucleotides were synthesized by Integrated DNA Technologies (Coralville, IA). A standard curve was generated by amplifying 1 µL aliquots of 10^8^, 10^7^, 10^6^, 10^5^, 10^4^, and 10^3^ serially diluted pBSHPV18 copy number controls. Acceptable *R*^2^ values for standard curves were at or above 0.99. A Bio-Rad CFX-96 Real-Time qPCR machine and software were utilized for PCR amplifications and subsequent data analysis. The PCR thermocycling profile was as follows: a 15-min hot-start at 95°C, followed by 40 cycles of 15 s at 94°C, 30 s at 52°C, and 30 s at 72°C. The data were collected during the extension phase.

### RT-qPCR infectivity assays

HaCaT cells were grown to confluence in Dulbecco’s modified Eagle medium supplemented with 10% fetal bovine serum, 2 mM glutamine, 1 mM pyruvate, 100 U of penicillin/mL, and 100 µg of streptomycin/mL and seeded at 50,000 cells/well in 24-well plates (16, 20, 21). Benzonase-treated HPV18 viral preparations were diluted with cell culture medium to a total volume of 0.5 mL. Medium was aspirated from HaCaT cells, and 0.5 mL of diluted viral prep was added per well. An MOI of 10 for non-fractionated virus and an MOI of 5 for fractionated virus were utilized for infections. For antibody-mediated neutralizations, an MOI of 10 was incubated with a 1:100 dilution of anti-L1 (18.K2 or 18.J4) or anti-L2 (RG-1) antibody for 1 h at 37°C prior to infection. One well on each plate received 0.5 mL of medium without virus as a negative control. The cells were incubated with the virus for 48 h at 37°C. mRNA was harvested using the RNeasy kit (Qiagen). Amplification of both the viral target and the endogenous cellular control target was performed by using a duplex format in 0.2 mL, 96-well PCR plates (Bio-Rad). All reactions containing RNAs from virus-infected cells were performed in duplicate. RT-qPCR was performed in the same closed tube with ~250 ng of total RNA per reaction using the Quantitect Probe RT-PCR kit (Qiagen). The HPV18 E1^E4 primers used were as follows: forward 5′-GCTGATCAGAACCAGTGA-3′ and reverse 5′-CTGGCGTAGTCTTGCGTG-3′ at final concentrations of 4 µM. A fluorogenic, dual-labeled, HPV18-specific E1^E4 probe (5′-6-FAM-CCTCACCGTATCAGCACGTGTCGTG/3BHQ-1/-3′) was utilized at a final concentration of 0.2 µM to detect E1^E4 cDNA. TATA-binding protein (TBP) was amplified as a control using the primers as follows: 5′-CACGGCACTGATTTTCAGTTCT-3′ and 5′-TTCTTGCTGCCAGTCTGGACT-3′ at final concentrations of 0.125 µM. All primers and probes were synthesized by Integrated DNA Technologies (Coralville, IA). All RT-qPCR reactions were performed using the CFX-96 (Bio-Rad). Cycling conditions were 50°C for 30 min (RT) and 95°C for 15 s, followed by 42 cycles of 94°C for 15 s and 54.5°C for 1 min. The relative quantities of viral target cDNA were determined using REST software.

### Immunoblot analysis

Aliquots of Optiprep-purified virus were boiled for 10 min in 6% 2-mercaptoethanol (2-ME) loading buffer and loaded onto a 7.5% polyacrylamide gel. Proteins were then transferred to a nitrocellulose membrane and blocked using SuperBlock blocking buffer in PBST (Thermo Scientific). Membranes were incubated overnight with primary antibodies. Monoclonal antibodies against HPV18 L1, 18.L9, and 18.E20 were used at a dilution of 1:500. Biotin-Goat Anti-Mouse IgG (H+L) (Invitrogen) was used at a dilution of 1:10,000, followed by Streptavidin Horseradish Peroxidase (HRP) Conjugate (Invitrogen) at a dilution of 1:5,000. Membranes were washed with PBST after the addition of each antibody. All antibodies were diluted in SuperBlock blocking buffer. The proteins were detected using an ECL kit (Perkin Elmer).

### Disinfection treatment to determine virion stability

Each disinfectant was prepared according to the manufacturer’s recommendation and stated dilution: 0.525% hypochlorite and 0.55% OPA. The disinfectant was mixed thoroughly with virus and incubated for 45 min at room temperature. Following incubation, a neutralizer (0.1% Tween 80, 1% peptone, 1% cysteine, and 0.5 M Tris buffer, pH 7.5) was added to the virus/disinfectant, and the mixture was centrifuged in an Amicon Ultra centrifugal filter (100,000 molecular weight cutoff; Millipore) at 4,000 rpm for 10 min. Additional neutralizer was added, and the columns were once again centrifuged at 4,000 rpm for 10 min. Two additional washes with HaCaT culture medium, prepared in accordance with the methods previously described (21), were performed. The resulting liquid containing the treated virus was collected, and the virus was assayed for infectivity, as described above. In this study, we used a method in line with U.S. Food and Drug Administration (FDA) guidelines for assessing virion stability when treated with different disinfectants (22).

### Denatured ELISA

Virus preparation (30 µL) and denaturant (20 µL) were incubated in a 96-well round-bottom plate (Evergreen) at 37°C overnight in a humidified incubator. Wells were blocked with SuperBlock in PBST and incubated with monoclonal antibodies against L1, 18.L9, or 18.61-26 at a dilution of 1:500. For detection, para-nitrophenyl phosphate (PNPP) was used. Samples were incubated at room temperature until color developed and were read at 405 nm using a plate reader. A control experiment for this assay using HPV16 and HPV18 wild type can be found in Fig. S5.

## RESULTS

### Impact of upstream Met residues on virus infectivity

**Fig 2:**
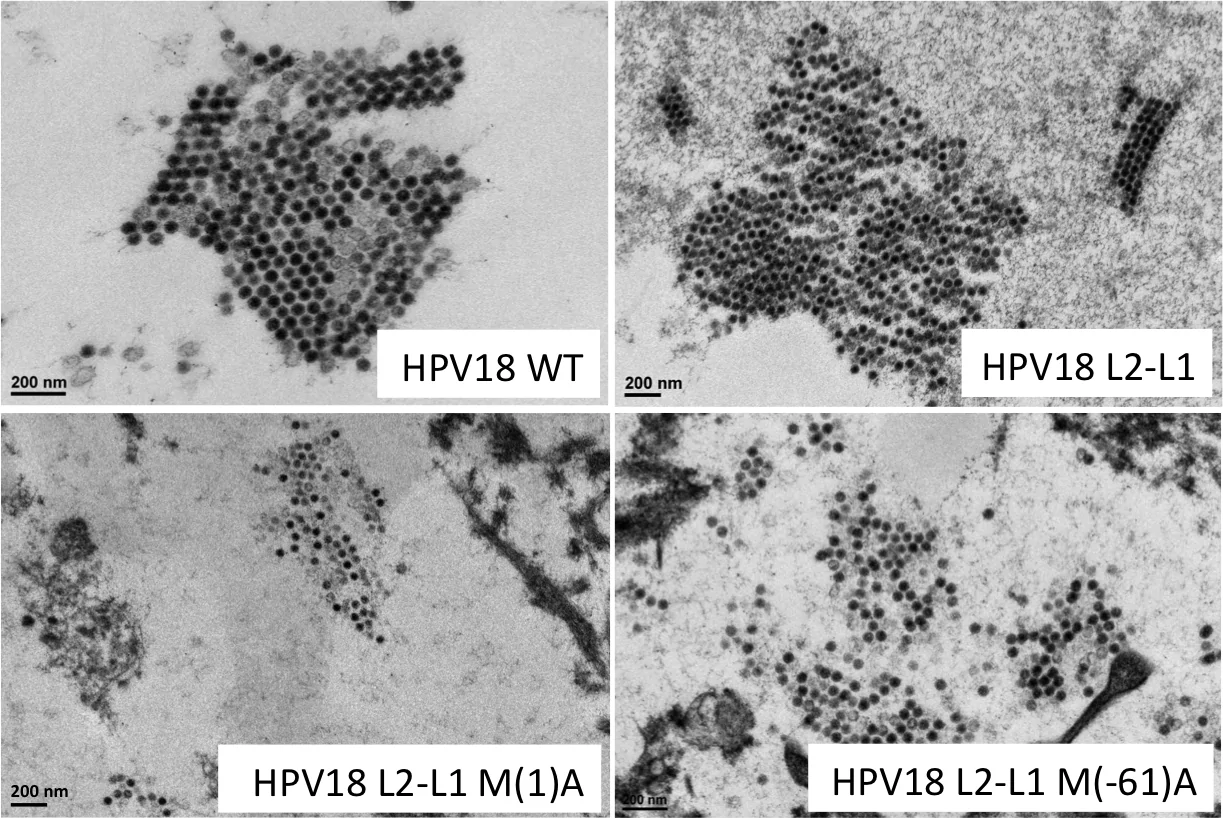
Production of native HPV18 virions with and without the upstream in-frame methionines. TEM examination of raft cultures from cell lines that consistently maintain episomal copies of HPV18 WT, HPV18 L2-L1, HPV18 L2-L1 M(1)A, and HPV18 L2-L1 M(−61)A and produce virus.

To determine whether M(−61) and/or M(−26) are important to the viral life cycle of HPV18, single, double, or triple substitution mutants were generated, replacing the L1 N-terminal Met residues with alanine (Fig. S1). Since the ORFs of L1 and L2 overlap, we created a HPV18 genome, named HPV18 L2-L1 (L2-L1), in which the L1 and L2 capsid ORFs are separated (Fig. 1B) (14). This allowed for the mutation of the L1 N-terminus without creating mutations in the L2 C-terminus. Primary human foreskin keratinocytes (HFKs) were electroporated with wild-type and mutant genomes to create immortalized cell lines that stably maintained episomal copies of the HPV18 genome. These cell lines were grown in organotypic raft culture for 20 days to produce fully mature virions (16). All cell lines grew and differentiated into organotypic raft cultures that were consistent with wild-type HPV18 tissue morphology (Fig. S2). Electron microscopy (EM) was used to confirm the presence of intact virions in tissue (Fig. 2).

**Fig 3:**
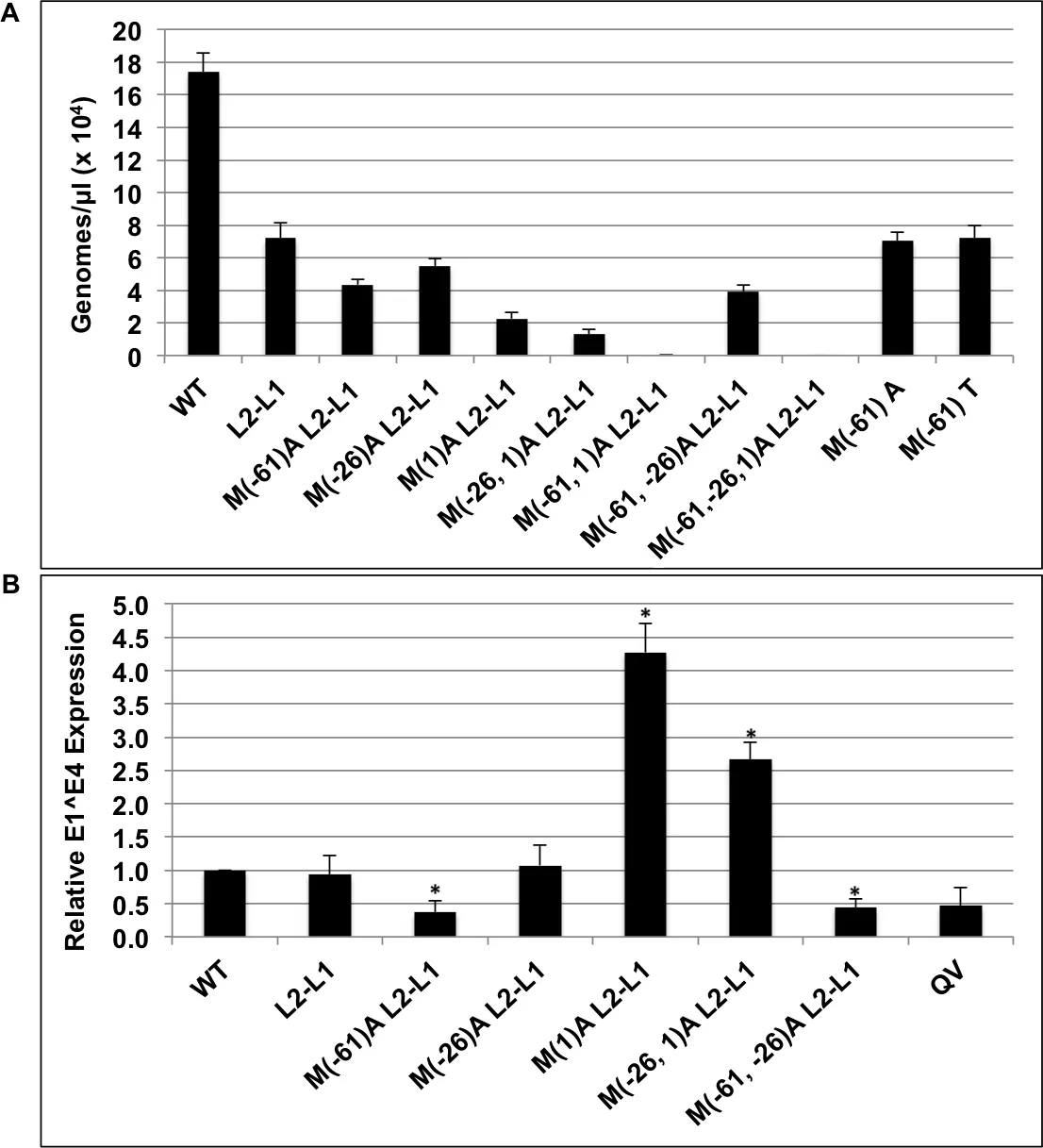
The ablation of upstream in-frame L1 methionines impacts the infectivity of HPV18. (**A**) Titration of wild-type or mutant virions produced in organotypic raft culture from human foreskin tissue. Tissue containing virus was Dounce homogenized to generate a virus preparation. Viral titer was determined by quantitating the number of encapsidated viral genomes against a standard curve of known HPV DNA concentrations in a qPCR assay. (**B**) Infection of HaCaT cells by HPV18 wild type, HPV18 Met mutants, and HPV18 QV (MOI = 10). Forty-eight hours post-infection, mRNA was harvested, and the levels of the E1^E4 splice transcript were determined by qRT-PCR. The levels of infection are shown relative, with wild type being set equal to 1. This figure is representative of at least three individual experiments using at least two different virus preparations for each virus type. Bars represent standard deviation. An asterisk (*) denotes significance by Student's *t*-test. Statistical significance was defined at *P* ≤ 0.05.

Mutations in L1 did not impact viral DNA replication within the tissue, as similar levels of DNA replication were seen for wild-type and the mutant HPV18 genomes observed using Southern blotting analysis (Fig. S3). However, as expected, there was an effect on virion production (Fig. 3A).

Viral infectivity was assessed by infecting HaCaT cells with equal titers (MOI of 10) of wild-type and mutant viruses. HPV18 QV, generated from an expression vector with L1 translation restricted to M(1), was used for comparison (17) as QV resembles native virion and can be utilized to study various aspects of HPV structure, assembly, and maturation. Infectivity was determined 48 h post-infection by measuring the relative amounts of E1^E4 splice transcript, a viral transcript found abundantly in infected cells (Fig. 3B) (16). Viruses from wild type and from the genome with separated capsid protein ORFs, L2-L1, were equally infectious. The M(−26)A L2-L1 mutant had a similar level of infectivity compared to wild type. Both the M(−61)A L2-L1 mutant and the QV were more than 50% less infectious than wild type. In contrast, the M(1)A L2-L1 mutant was more than 4-fold more infectious than wild type. Double mutants with methionine-to-alanine substitution showed similar results (Fig. 3B). The M(−26,1)A L2-L1 double mutant, with alanine substitutions at M(−26) and M(1), displayed an increased infectivity phenotype similar to the M(1)A L2-L1 mutant. The M(−61,−26)A L2-L1 double mutant showed a similar decrease in infectivity compared to M(−61)A L2-L1 and QV. We were unable to measure infectivity for the M(−61,1)A L2-L1 double mutant and the M(−61,−26,1)A triple mutant, as no virions were detected (Fig. 2A). Transfection of equal amounts of wild-type and mutant viral genomes susceptible to endonuclease into HaCaT cells confirmed that mutations did not negatively impact E1^E4 production, as all mutant viral genomes tested produced equal amounts of the viral transcript 48 h post-transfection compared to wild type (Fig. S4). These experiments suggest that in the L1 N-terminus, M(−61) and M(1), but not M(−26), are used to synthesize virions and play an important role in HPV18 infection.

### Capsid stability of virions harboring mutations in upstream Met residues in L1

**Fig 4:**
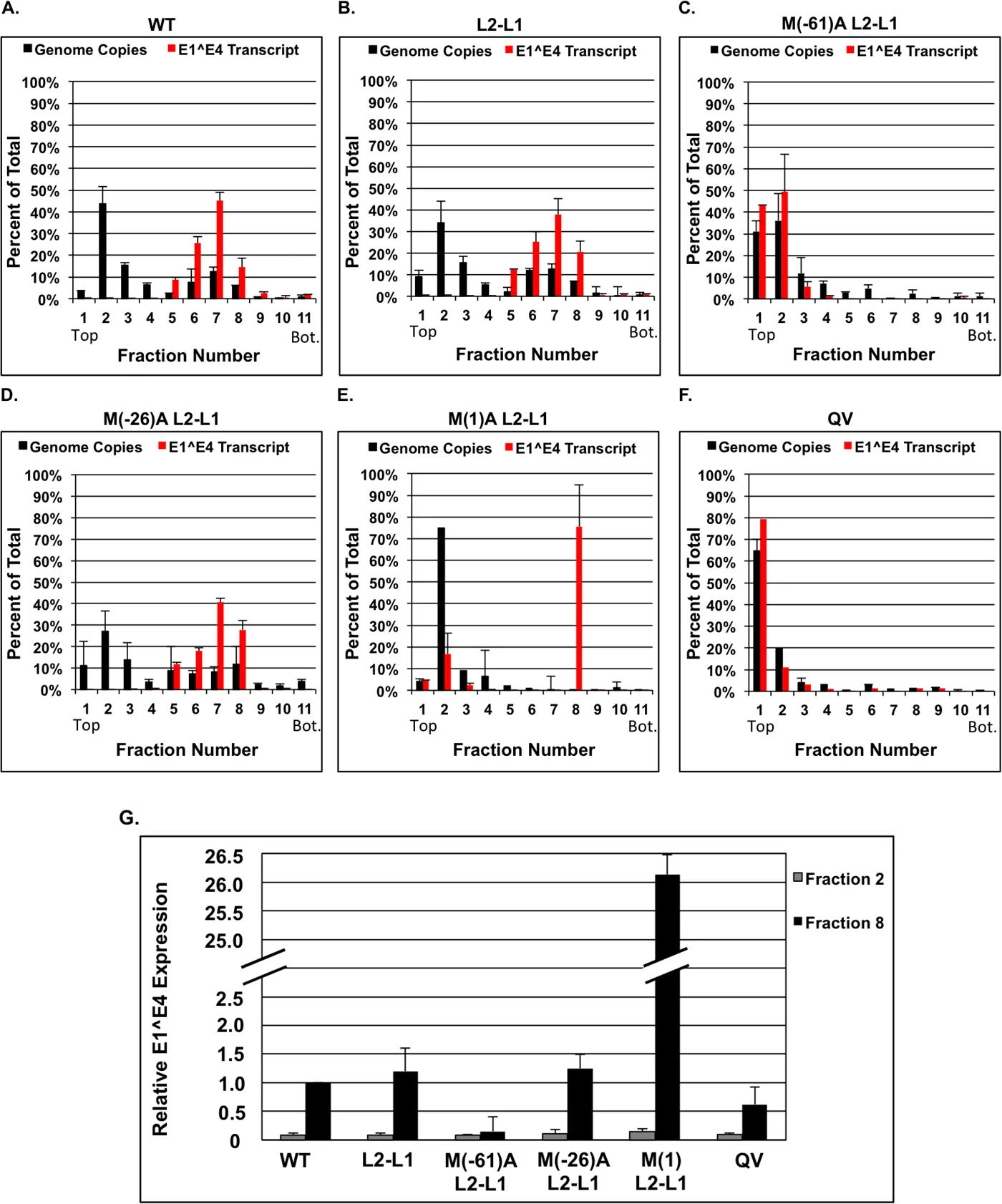
Mutation of M(−61) and M(1) reduces capsid stability in HPV18. (**A–F**) HPV18 wild type, mutant HPV18 virions, and HPV18 QV were added onto an OptiPrep gradient and subjected to ultracentrifugation. Eleven 500 μL fractions were taken from each gradient, with fraction 1 being the top and fraction 11 being the bottom of the gradient. Each fraction was titered and tested for infectivity. Genome quantification by qPCR is shown as the percentage total genome copies (black bars). Infectivity was tested by infecting HaCaT cells with an equal volume (50 μL) of virus from each fraction. Forty-eight hours post-infection, mRNA was harvested from infected cells, and the E1^E4 splice transcript was detected in a RT-qPCR-based assay as a measure of infectivity. Infection is shown as percent total of E1^E4 transcript detected (red bars). (**G**) HaCaT cells were infected at an MOI of 5 with each virus from fraction 2 (free DNA – gray bars) or fraction 8 (purified virus – black bars). Forty-eight hours post-infection, mRNA was harvested, and relative levels of the E1^E4 splice transcript were measured by RT-qPCR as a measure of infectivity. All figures are representative of at least three individual titrations or infections using at least two different virus preparations derived from at least two different sets of rafts. Bars represent standard deviation.

Previous studies have demonstrated that HPV mutants that have altered infectivity often exhibit altered capsid stability (19–21). Our laboratory has seen this most recently with HPV16 mutants that negatively impact critical disulfide bonds within the capsid (17). Investigation of the capsid stability was performed as described previously (16). Briefly, the virions pre-treated with endonuclease were subjected to ultracentrifugation, which has previously been reported to make viral genomes susceptible to endonuclease digestion more frequently if a capsid is unstable (16). After ultracentrifugation, 11 fractions were taken (1 = top and 11 = bottom). Total genome copies were measured by qPCR within each fraction and were expressed as percent genome copies compared to the total number of genome copies among all fractions. Fractions 1–4 contained genomes that were not protected from endonuclease post-centrifugation, suggesting that they were dissociated from their capsid or were naked genomes (16). Since virions were treated with endonuclease before fractionation, genomes detected in fractions 1–4 became unprotected from endonuclease due to ultracentrifugation, rather than being previously in that state pre-centrifugation (16). Fractions 6–9 contained genomes that were inside protected capsids and were still resistant to endonuclease post-centrifugation (16). Wild type, L2-L1, and M(−26)A L2-L1 showed similar genome copy fractionation profiles, with almost half of their genomes detected in the most stable fractions 6–9 (Fig. 4A–B , and D).

M(−61)A L2-L1, M(1)A L2-L1, and QV showed similar genome fractionation profiles, with most of their genome copies detected in the less stable fractions 1–4 (Fig. 4C, E and F). To quantify the differences, relative stabilities were calculated, defined as the ratio of total unprotected genomes to total protected genomes (23). The lower the number, the more stable the population of virions. The relative stabilities of HPV18 wild type, L2-L1, and M(−26)A L2-L1 were similar and very close to those reported for mature HPV16 wild type (Table 1) (16).

**TABLE 1:** Relative stability values of Met mutants and data summation^*a*^

Virus type	Infectivity^*b*^	Relative stability	Neutralization	L1 size detected
WT	++	2.5	RG-1, K2, J4	Small, large (54, 55 kDa)
L2-L1	++	1.9	RG-1, K2, J4	Small, large (54, 55 kDa)
M(−61)A L2-L1	+	10.4	RG-1, J4	Small (54 kDa)
M(−26)A L2-L1	++	1.9	RG-1, K2, J4	Small, large (54, 55 kDa)
M(1)A L2-L1	+++	47.3	None	NA
QV	+	12.7	RG-1 (varied), J4	Smallest (53 kDa)
M(−61)A	+	11.8	RG-1, J4	Small (54 kDa)
M(−61)T	+	8.2	RG-1, J4	Small (54 kDa)

^*a*^ Relative stability values were calculated by quantifying the ratio of total unstable genomes to total stable genomes. The lower the number, the more stable the population of virions. Data are summarized for all wild type, mutant viruses, and QV for infectivity, stability, antibody neutralization, and protein size detected by western blot. Following Optiprep gradient ultracentrifugation, no viable M(1)A L2-L1 virus remained (NA). M(−61)A L2-L1 and QV were each approximately five times less stable than the wild type. The M(1)A L2-L1 mutant was approximately 20 times less stable than wild type, as following Optiprep gradient ultracentrifugation no viable M(1)A L2-L1 virus remained. These findings indicate that M(−61) and M(1), but not M(−26), in HPV18 L1 play crucial roles in capsid stability.

^*b*^ WT infectivity is symbolized by ++, virus types which are less infective are denoted by +, and virus type more infective than WT is symbolized by +++.

To further examine the characteristics of the fractionated particles, total infectivity was analyzed for each fraction. Mature wild-type HPV16 virions have previously been shown to have most of their infectivity detected in fractions 6–9 (16). Here, the wild type, L2-L1, and M(−26)A L2-L1 also had most of their infectivity detected within these stable fractions (Fig. 4A, B and D). The M(−61)A L2-L1 mutant and QV had poor infectivity within the stable fractions, while residual infectivity was detected within the unstable fractions (Fig. 4C and F). The M(1)A L2-L1 mutant had detectable infectivity in the unstable fractions but had a repeatable spike of infectivity detected in fraction 8 (Fig. 4E). Comparing infectivity of fractions 2 and 8 for wild-type, L2-L1, and Met mutants at an MOI of 5 showed that genomes detected in the stable fractions were far more infectious than free genomes detected in non-stable fractions (Fig. 4G). Wild-type, L2-L1, and M(−26)A L2-L1 fraction 8 samples had similar levels of infectivity. The M(−61)A L2-L1 mutant and QV fraction 8 samples were both 50% or more less infectious than the wild type. The M(1)A L2-L1 mutant fraction 8 sample was over 26-fold more infectious than wild type. These experiments show that virions with M(−61) available for L1 translation are more infectious than those without. This suggests that there is an important role for L1 produced by this upstream Met in HPV18 during infection.

### Virion capsid conformation following Met mutation

**Fig 5:**
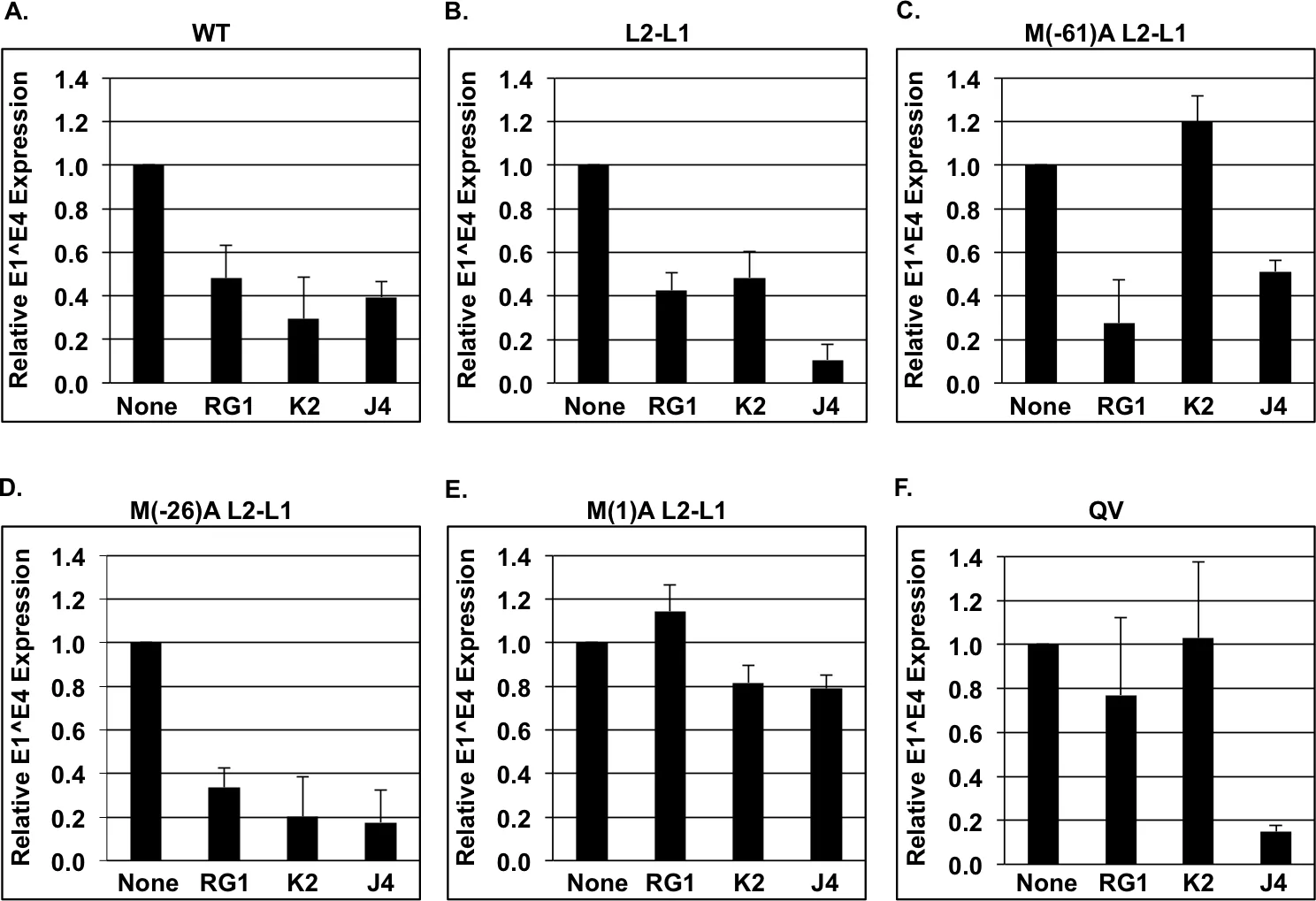
Mutation of M(−61) and M(1) changes capsid conformational epitopes. (**A–F**) Neutralizing monoclonal antibodies specific for L1 (J4 and K2) or L2 (RG-1) were incubated with virus (MOI = 10) prior to and during infection at a dilution of 1:100. Relative levels of E1^E4 spliced transcript were detected by RT-qPCR in HaCaT cells 48 h post-infection. Each experiment was conducted in triplicate. Error bars represent standard deviation.

Antibodies have been used to distinguish changes in the capsid conformation of HPV particles in various studies (16, 23). To determine if capsid conformation impacted the infectivity and stability differences of the mutants, both L1 (K2 and J4) and L2 (RG-1) neutralizing antibodies were incubated with the virus prior to and during infection. Neither of the HPV18 L1-specific monoclonal antibodies have been mapped; however, as they were produced using HPV18 VLPs, the epitopes must be downstream of M(1) on the L1 protein. Wild-type, L2-L1, and the M(−26)A L2-L1 mutants were all neutralized by K2, J4, and RG-1 monoclonal antibodies, suggesting a native capsid conformation (Fig. 5A, B and D). The M(−61)A L2-L1 mutant was neutralized by J4 and RG-1, but it could not be neutralized by the K2 antibody (Fig. 5C). This is consistent with a similar loss of K2 neutralization previously observed in a mutant that had the portion of L1 from M(−61) up to M(−26) deleted (14). The QV also was not neutralized by the K2 antibody and showed varying neutralization to RG-1 (Fig. 5F). The M(1)A L2-L1 mutant lost neutralization to all three antibodies, suggesting a highly altered capsid structure (Fig. 5E). Loss of neutralization with conformation-dependent L1 antibodies has been previously seen in hybrid HPV16 VLPs where portions of L1 were replaced with L1 from another HPV type (24). This evidence further supports that M(−61) and M(1) have a crucial role in the capsid structure of the HPV18 virion, as mutants which have lost one of these Met residues exhibit capsid conformational differences compared to wild type. This detected change in capsid conformation could explain the altered stability and infectivity observed in mutants M(−61)A L2-L1 and M(1)A L2-L1 as well as the QV preparation.

### L1 protein translation products from Met mutants

**Fig 6:**
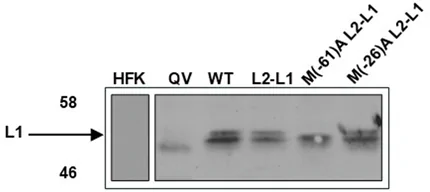
Mutation of M(−61) causes a loss of the larger size L1. Western blot analysis of fraction 8 samples of wild type, Met mutants, and QV. A fraction 8 sample from a primary HFK organotypic raft, which was HPV negative, was used as a negative control. The experiment was repeated three times.

We next determined whether the upstream Met residues in HPV18 contribute to the HPV life cycle by producing different sizes of L1 capsid proteins. L1 has been previously detected at approximately 55 kDa by western blot analysis (16, 20). Using the stable fraction 8, two L1 proteins migrating between 46 and 58 kDa were detected in wild-type, L2-L1, and the M(−26)A L2-L1 mutant, utilizing the H18.L9 monoclonal antibody (Fig. 6). This is similar to what has been detected in HPV16 (16). M(−61)A L2-L1 only showed the faster of the two migrating sizes of L1 protein found in wild type. This suggests that the slower-migrating size of L1 is translated from M(−61). QV showed a single L1 size that migrated faster than either of the bands produced in organotypic rafts (Fig. 6). It is possible that this difference in migration is caused by a lack of post-translational modification, which has often been described to occur with L1 derived from differentiated tissue (25, 26).

The L1 protein from the M(1)A L2-L1 mutant could not be visualized (data not shown), even after sample concentration and extended periods of exposure. It is possible that, due to the low number of encapsidated genomes quantified post-centrifugation (Fig. 4E), L1 could not be detected in the stable fraction 8 of the M(1)A L2-L1 mutant. No bands between 46 and 58 kDa were detected in an HPV-negative primary HFK organotypic raft sample using the anti-L1 antibody. The western blot experiments suggest that HPV18 wild type produces two forms of L1 capsid protein, with the longer version translated from M(−61) and the shorter version translated from M(1).

### Biological impact of upstream Met residues on wild-type genome

**Fig 7:**
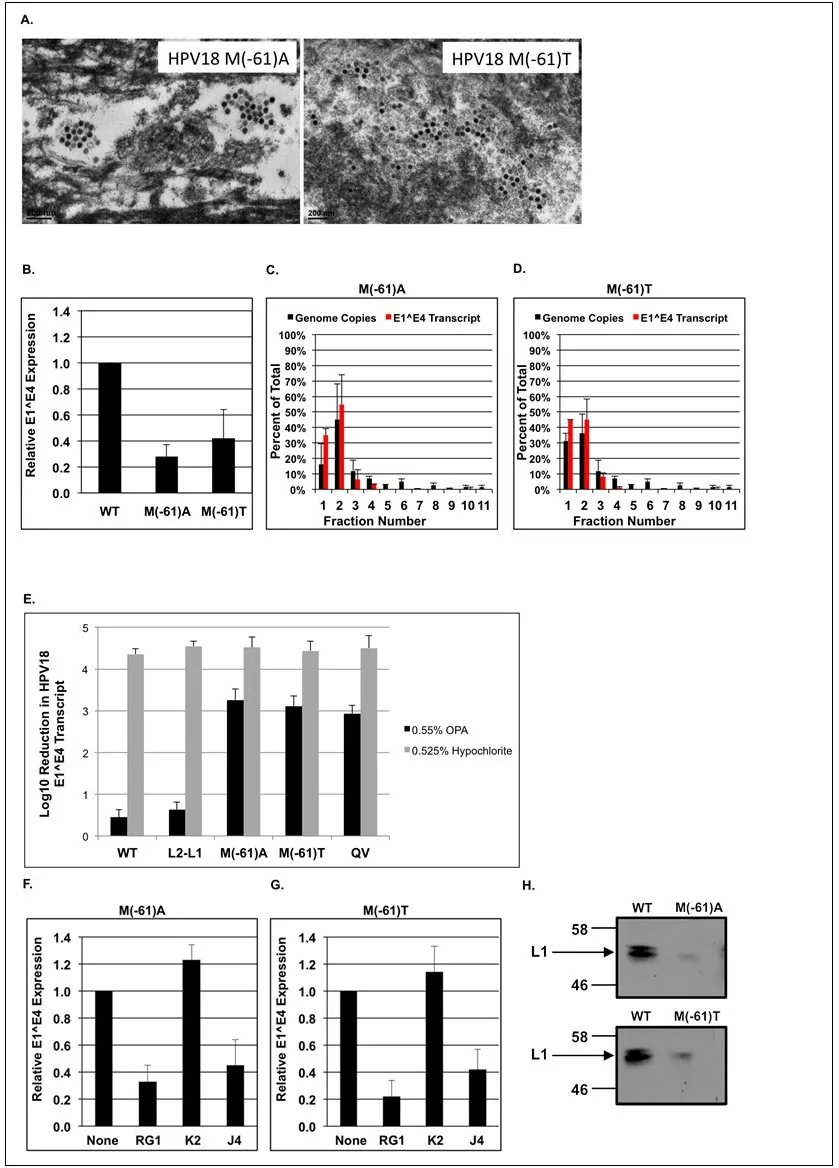
Mutating M(−61) causes a similar viral phenotype in an HPV18 genome with a native capsid ORF configuration. (**A**) TEM examination of HPV18 M(−61)A-and HPV18 M(−61)T-infected raft cultures. (**B**) Relative levels of E1^E4 spliced transcript detected by RT-qPCR in HaCaT cells 48 h post-infection (MOI = 10). Statistical significance was defined at *P* ≤ 0.05. (**C and D**) Percent total genome copies and infectivity were measured after fractionating viral preps in an ultracentrifuge. Equal volumes of each fraction were used to measure total genome copies per fraction detected using qPCR (black bars). Equal volumes of each fraction were used to measure total infectivity per fraction by measuring E1^E4 spliced transcript detected by RT-qPCR in HaCaT cells 48 h post-infection (red bars). (**E**) Infection of cells with disinfectant-treated virus. Quantitation of the E1^E4 splice transcript in a RT-qPCR based assay was utilized as a measure of infectivity. Post-infection, infectivity was compared to input to determine the log10 reduction in infectivity. Each experiment was conducted in triplicate. Error bars are representative of standard deviation. (**F and G**) Neutralizing monoclonal antibodies specific for L1 (J4 and K2) or L2 (RG-1) were incubated with virus (MOI = 10) prior to and during infection at a dilution of 1:100. Relative levels of E1^E4 spliced transcript were detected by RT-qPCR in HaCaT cells 48 h post-infection. Statistical significance was defined at *P* ≤ 0.05. (**H**) A western blot was performed using fraction 8 samples of wild-type and Met mutants.

It is unknown how the separation of the capsid ORFs in L2-L1 may affect L1 translation (Fig. 1B), so the mutation of M(−61)A was created in the HPV18 wild-type genome. This mutation also changes one amino acid in the C-terminus of L2 from asparagine to glycine (nt 5,430–5,432). A second mutant, M(−61)T, was also made, as this mutation leaves all amino acids in-frame with the L2 ORF unchanged. Histological staining for the E4 protein of organotypic rafts showed similar levels and areas of viral expression between wild type and mutants within the native capsid ORF configuration (Fig. S5). Production of intact virions for both the M(−61)A and M(−61)T mutants was confirmed by EM (Fig. 7A).

Both the M(−61)A and M(−61)T mutant viruses were 50% less infectious than wild type (Fig. 7B), similar to the M(−61)A L2-L1 mutant (Fig. 7A). Upon fractionation, a majority of the genomes detected appeared in the less stable fractions (Fig. 7C and D), with M(−61)A and M(−61)T having relative stabilities of 11.8 and 8.2, respectively (Table 1). The M(−61)A L2-L1 mutant (Fig. 4C) shares the fractionation profile of M(−61)A and M(−61)T, as well as a similar relative stability of 10.4 (Table 1). We have previously published studies showing susceptibility of native HPV virions and QV stability to various clinical disinfectants (27, 28). To assess virion stability, we evaluated the susceptibility of Met mutants, L2-L1 mutants, quasivirus, and wild-type HPV18 to clinical disinfectants, including 0.525% hypochlorite (bleach) and 0.55% ortho-phthalaldehyde (Cidex OPA). As expected, both wild-type HPV18 and the L2-L1 mutant were susceptible to disinfection by 0.525% hypochlorite, but not by 0.55% OPA (Fig. 7E) (27, 28). In contrast, the M(−61)A and M(−61)T mutants, such as HPV18 QV, were susceptible to disinfection by both 0.525% hypochlorite and 0.55% OPA, indicative of less stable virions (Fig. 7E). We showed that M(−61)A and M(−61)T could be neutralized by J4 and RG-1, but not by K2 (Fig. 7F–G), similar to the M(−61)A L2-L1 mutant (Fig. 5C). Both mutant viruses also show only the shorter L1 on a western blot, similar to the M(−61)A L2-L1 mutant (Fig. 7H).

**Fig 8:**
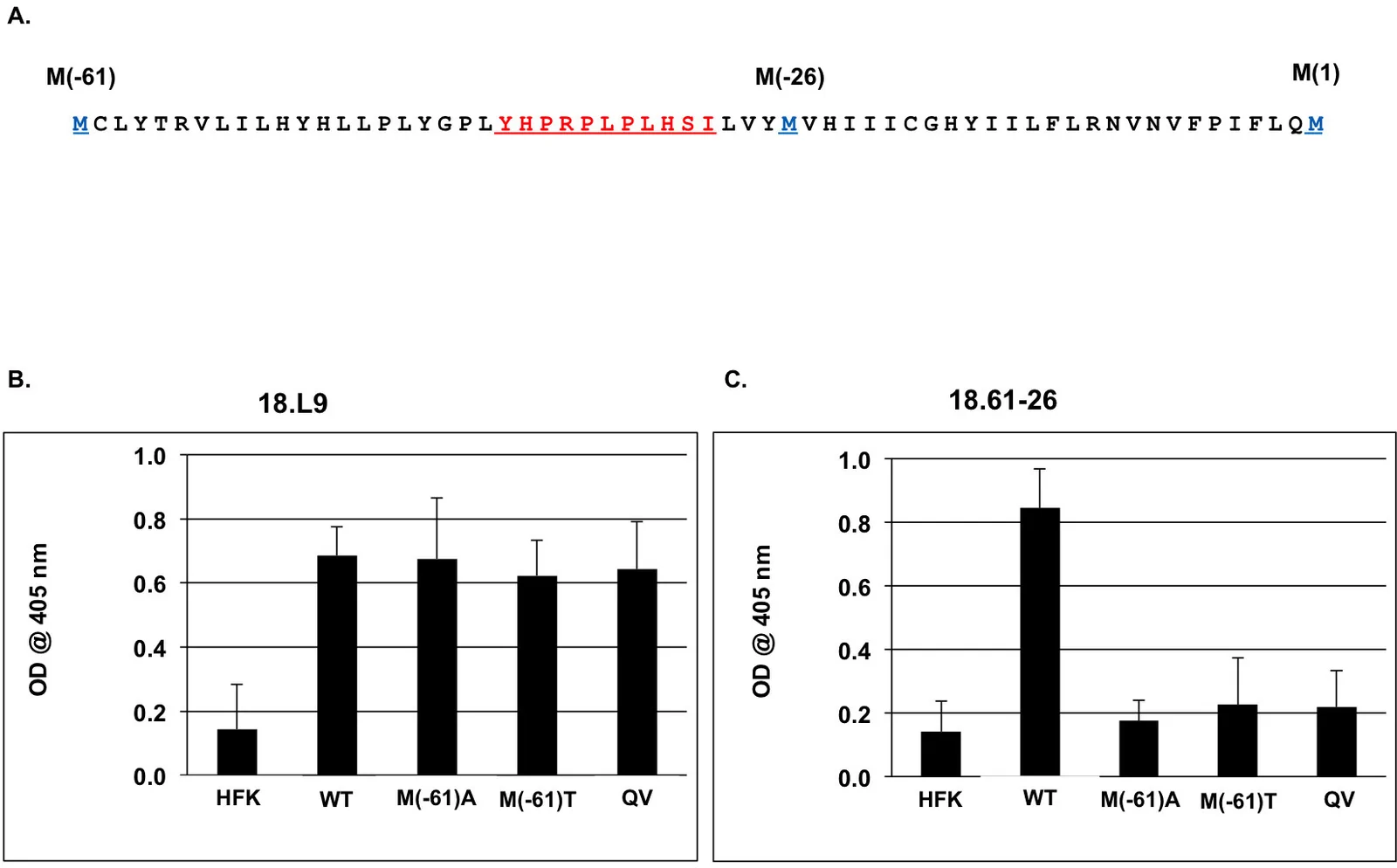
Differential reactivity of 18.61-26 polyserum with wild-type and mutant HPV18 preparations. (**A**) Amino acid map of M(−61) to M(1) in HPV18 L1. Highlighted are the three methionines (blue) and the peptide sequence (red) used to create the 18.61-26 polyserum. (**B and C**) HPV18 wild type, M(−61)A, and M(−61)T were incubated with denaturant. HPV18 L1 total (18.L9) or only the large isoform (18.61-26) was detected at 405 nm.

To further confirm our findings, an immunogenic region between M(−61) and M(−26) was synthesized as an immunizing peptide and used to produce polyserum (18.61–26) in rabbits that would detect the larger form of L1 (Fig. 8A). In a denatured ELISA, this polyserum showed preferential reactivity with wild-type HPV18 preparations, while QV, M(−61)A, and M(−61)T preparations showed signals near background levels (Fig. 8C). To show specificity of the assay, HPV16 and HPV18 viruses were incubated with type-specific L1 monoclonal antibodies 18.L9 (HPV18) and Camvir (HPV16) (Fig. S6). Additionally, as a control, monoclonal antibody raised against HPV18 VLPs (18.L9) confirmed the presence of L1 in all viral samples (Fig. 8B). These findings are consistent with the presence of an N-terminally extended L1 species in wild-type HPV18 virions.

## DISCUSSION

Most experimentation performed on HPV structure has been performed using particles produced in monolayer cell culture systems. While these studies have yielded important scientific findings, including the platform to create a working vaccine strategy targeting HPV infection and cervical cancer, it is still unclear what differences exist among VLPs, PsVs, QVs, and native virions. We used the organotypic raft culture system for these studies to evaluate the mutants, alongside quasivirus. Organotypic raft culture systems helped to assess the mutants in a system that closely mimics the naturally differentiating human epithelium, which is essential for HPV replication *in vivo*. Our extensive experience with raft cultures has been instrumental in investigating the HPV life cycle and the replication of infectious viruses. Additionally, our recent publications have emphasized notable differences in the replication and maturation of native viruses in differentiating host tissue compared to those generated using quasivirus technologies (15, 18).

This study explored if the upstream in-frame methionines found in HPV18, but not included in the L1 expression vectors used to produce recombinant particles, are necessary in the natural life cycle of HPV18. HPV18 has two upstream Met residues, M(−61) and M(−26); thus, it was used as an exploratory model. We have explored mechanisms of infectivity, neutralization, and cross-neutralization epitopes, as well as disinfectant and microbicide sensitivity for all Met mutants, quasivirus, and wild type.

By substituting the upstream Met with alanine in the genome with separated capsid ORFs, we found that the M(−61)A L2-L1 mutant genome produces virions that are less infectious and less stable than wild type. Interestingly, HPV18 wild-type virus contains two sizes of L1 detected on a western blot, while the M(−61)A L2-L1 mutant only produces the faster migrating L1 species. This indicates that the larger size L1 originates from M(−61), which was previously thought not to be used as a translation start site within the HPV18 genome. Unfortunately, we could not detect any L1 bands from the M(1)A L2-L1 mutant, presumably because of the lower titer available post-fractionation. However, the M(−26)A L2-L1 mutant shows both sizes of L1 found in wild type, suggesting that the faster migrating band can be linked to M(1). The QV, our recombinant virus control that contains only L1 translated from M(1), appears to migrate faster than the smaller size L1 found in wild type. Previous reports have shown that papillomaviruses taken from a differentiating epithelium contain multiple forms of L1 that are either potentially post-translationally modified or represent different isoforms of L1 (25, 26, 29, 30). It is unknown whether or not native HPV18 L1 is post-translationally modified and if that explains the migration difference between M(1) L1 translated in monolayer versus differentiating epithelium. M(−61)A L2-L1 loses the ability to be neutralized by the anti-L1 monoclonal antibody, K2. A similar HPV18 deletion mutant, which does not include M(−61), is also not neutralized by K2 (14). This deletion in HPV18 L1 can produce HPV18/16 chimeric mutants in the presence of HPV16 L2. Without this deletion, infectious chimeric virus cannot be made, potentially because the region in L1 between M(−61) and M(−26) interferes with chimera virion formation (14). This also supports that this region is included in mature, native HPV18 virions. We suggest that this conformation change detected by antibody neutralization assays is associated with the physical change that causes both the decrease in stability and infectivity (31). It is important to note that silencing M(−61) in the L2-L1 genome produces the same phenotype as with silencing M(−61) in the HPV18 wild type. M(−61)T also has the same phenotype, which is important because this mutant does not alter the ability to produce infectious stocks or the function of the L2-neutralizing antibody RG-1. We further confirmed our findings by producing novel rabbit polyserum with a peptide region found between M(−61) and M(−26). These findings are consistent with, but do not independently establish, the presence of an N-terminally extended L1 species in wild-type HPV18 virions.

In conclusion, these data are consistent with M(−61) being responsible for translating a larger L1 in HPV18. This protein is incorporated into the capsid and provides the virion with an altered structure compared with that of a virion made only with the shorter L1 translated from M(1). Further investigation into the mechanism of L1 translation is required. Mechanisms of non-canonical translation that have been previously published, such as internal ribosome entry, leaky scanning, non-AUG initiation, ribosome shunting, re-initiation, ribosomal frameshifting, and stop codon readthrough, would be candidates for testing (32–35). However, this finding with HPV18 may not be indicative of all HPV types that include upstream in-frame L1 methionines, as HPV1 and HPV2 isolated from differentiating epithelium were found to only have one species of L1 protein (36, 37). It is clear that both M(−61) and M(1) L1 need to be included in the final wild-type HPV18 virion to ensure that the particle is both highly stable and infectious. The apparent conservation of this genomic architecture is consistent with the extensive evolutionary history documented for HPV18 lineages (38). Recombinant particles have enough stability to form a particle, but this particle is less stable than the wild-type virus. It is possible that the L1 we detect, which is translated from M(−61), is critical to the survival of HPV18 against natural phenomena encountered *in vivo*. The inclusion of two different sizes of L1 in the final virion is a paradigm shift in the structural biology of papillomaviruses. Some other HPVs (HPV 16 and HPV 45) have upstream in-frame Met residues; it is possible that this alternative translation of L1 could have a similar function in other HPV types.

## Data Availability

All data presented in the article are available upon request.

## SUPPLEMENTAL MATERIAL

Supplemental material jvi.00167-26-s0001.pdf Fig. S1 to S6.

ASM does not own the copyrights to Supplemental Material that may be linked to, or accessed through, an article. The authors have granted ASM a non-exclusive, world-wide license to publish the Supplemental Material files. Please contact the corresponding author directly for reuse.
