# Supplementary material for: New form of HPV18 L1 found in native virions contributes to virion stability and infectivity
Source: J Virol. 2026 Sep 11;100(10):e00167-26. doi: 10.1128/jvi.00167-26 (PMC13712748; doi:10.1128/jvi.00167-26)
Supplement: Supplementary file 1 [file jvi.00167-26-s0001.pdf]

Virus Type	Description
WT	Wild-type (no mutation)
L2-L1	L1 and L2 ORFs are separated
M(-61)A L2-L1	L2-L1 backbone; Met-Ala mutation at M(-61)
M(-26)A L2-L1	L2-L1 backbone; Met-Ala mutation at M(-26)
M(1)A L2-L1	L2-L1 backbone; Met-Ala mutation at M(1)
QV	Quasivirus (L1 expression vector including only M(1))
M(-26,1)A L2-L1	L2-L1 backbone; Met-Ala mutation at M(-26) and M(1)
M(-61,1)A L2-L1	L2-L1 backbone; Met-Ala mutation at M(-61) and M(1)
M(-61,-26)A L2-L1	L2-L1 backbone; Met-Ala mutation at M(-61) and M(-26)
M(-61,-26,1)A L2-L1	L2-L1 backbone; Met-Ala mutation at M(-61), M(-26), and M(1)
M(-61)A	Wild-type backbone; Met-Ala mutation at M(-61)
M(-61)T	Wild-type backbone; Met-Thr mutation at M(-61)

S1. Virus descriptions. Summary of wild-type, L2–L1 backbone, quasivirus, and site-directed mutant constructs, including the amino acid substitutions introduced at the indicated methionine initiation codons.

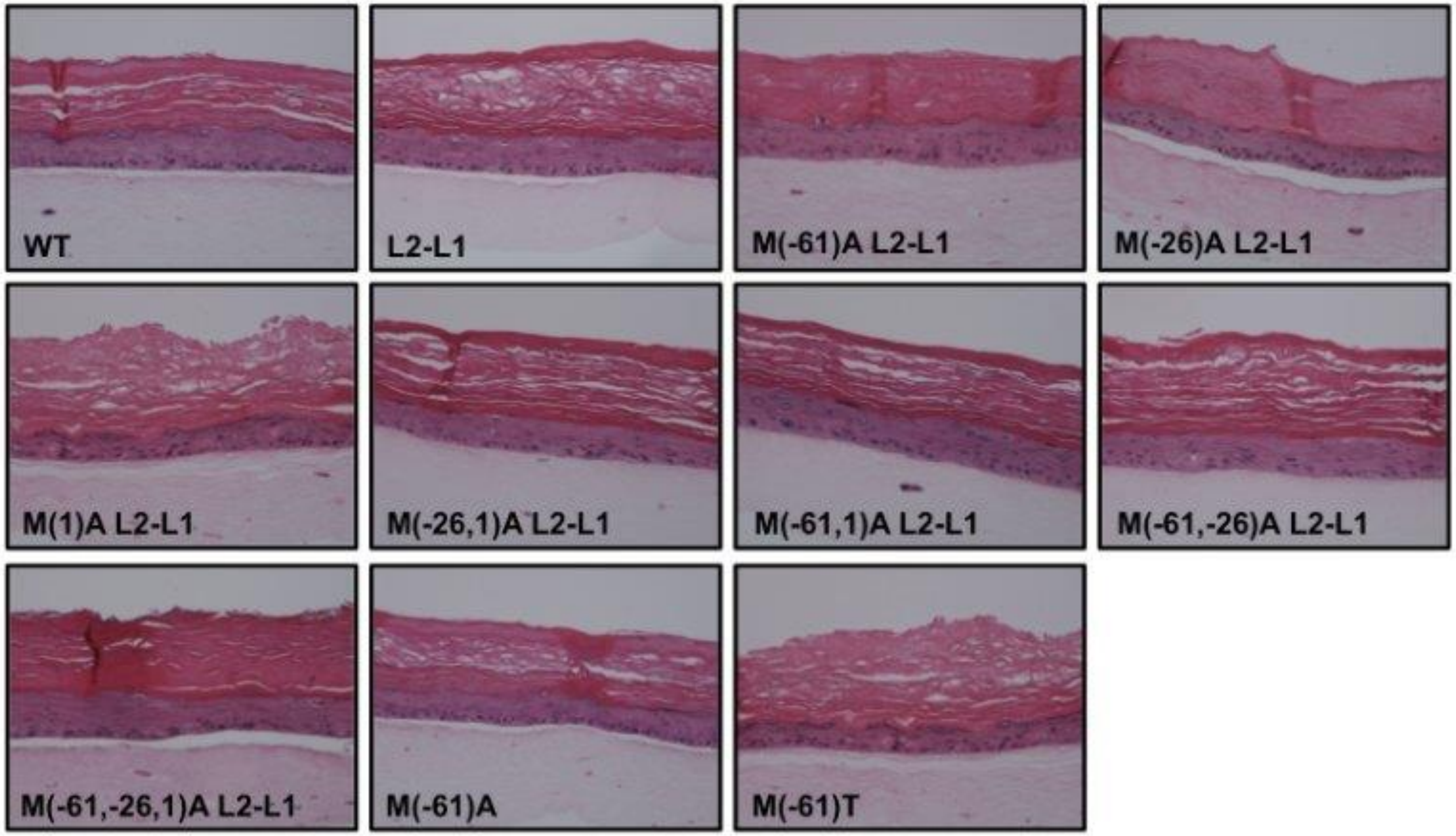

S2. Mutations do not inhibit organotypic raft growth. H&E staining of paraffin-embedded, wild-type and mutant raft tissues.

Virus Type	Fold Change in Amplification
Wild-Type	9.95
L2-L1	10.3
M(-61)A L2-L1	8.21
M(-26)A L2-L1	9.40
M(1)A L2-L1	8.95
M(-61)A	8.67
M(-61)T	8.84

S3. Mutations do not change the rate of viral amplification in differentiating tissue. Using q-PCR, the number of total virus genomes were quantified in 1 X 10^6 cells from wild-type or mutant cell lines before and after 20 days of raft growth. Amplification is defined as the ratio of genomes detected before and after 20 days of raft growth.

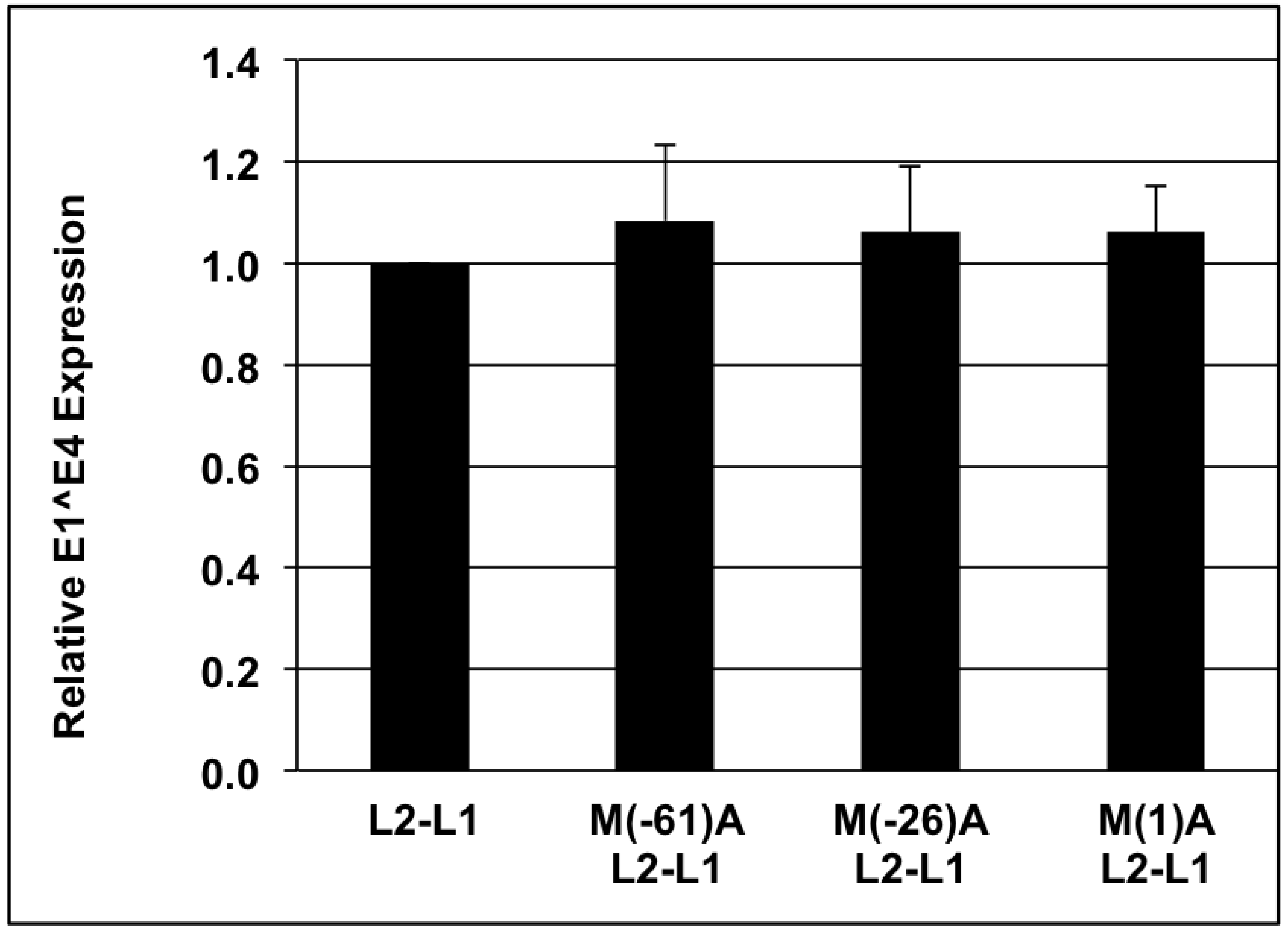

S4. Mutations do not change the amount of E1^E4 production in cells. L2-L1 and single Met mutant genomes were transfected into HaCaT cells at equal amounts. Shown are the relative levels of viral E1^E4 spliced transcript as detected by RT-qPCR 48 hours post-infection.

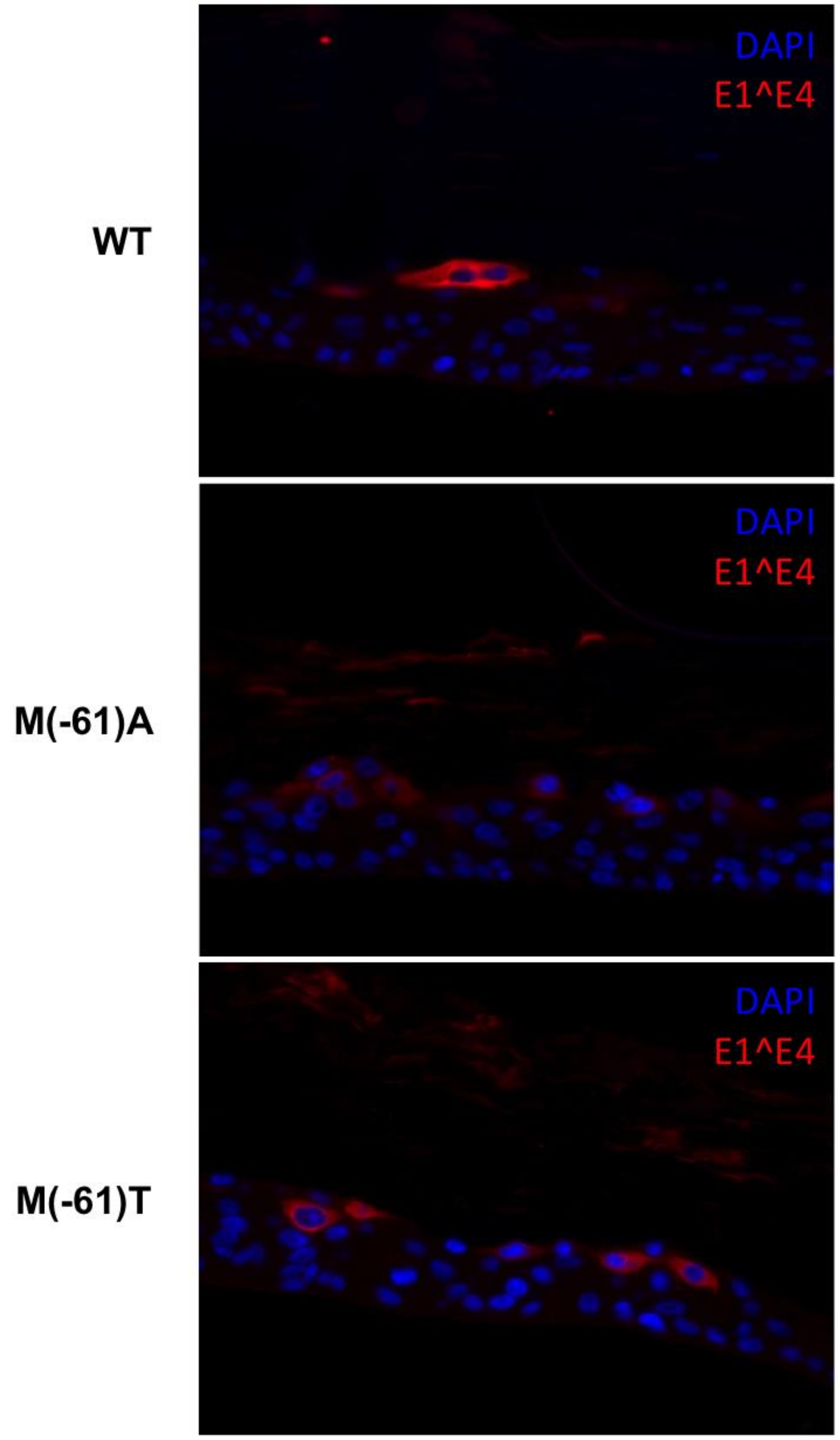

S5. E4 expression is unaffected by L1 M(-61) mutation. 20-day organotypic rafts of HPV18 wild-type, M(-61)A, and M(-61)T were sectioned and stained for E4 protein.

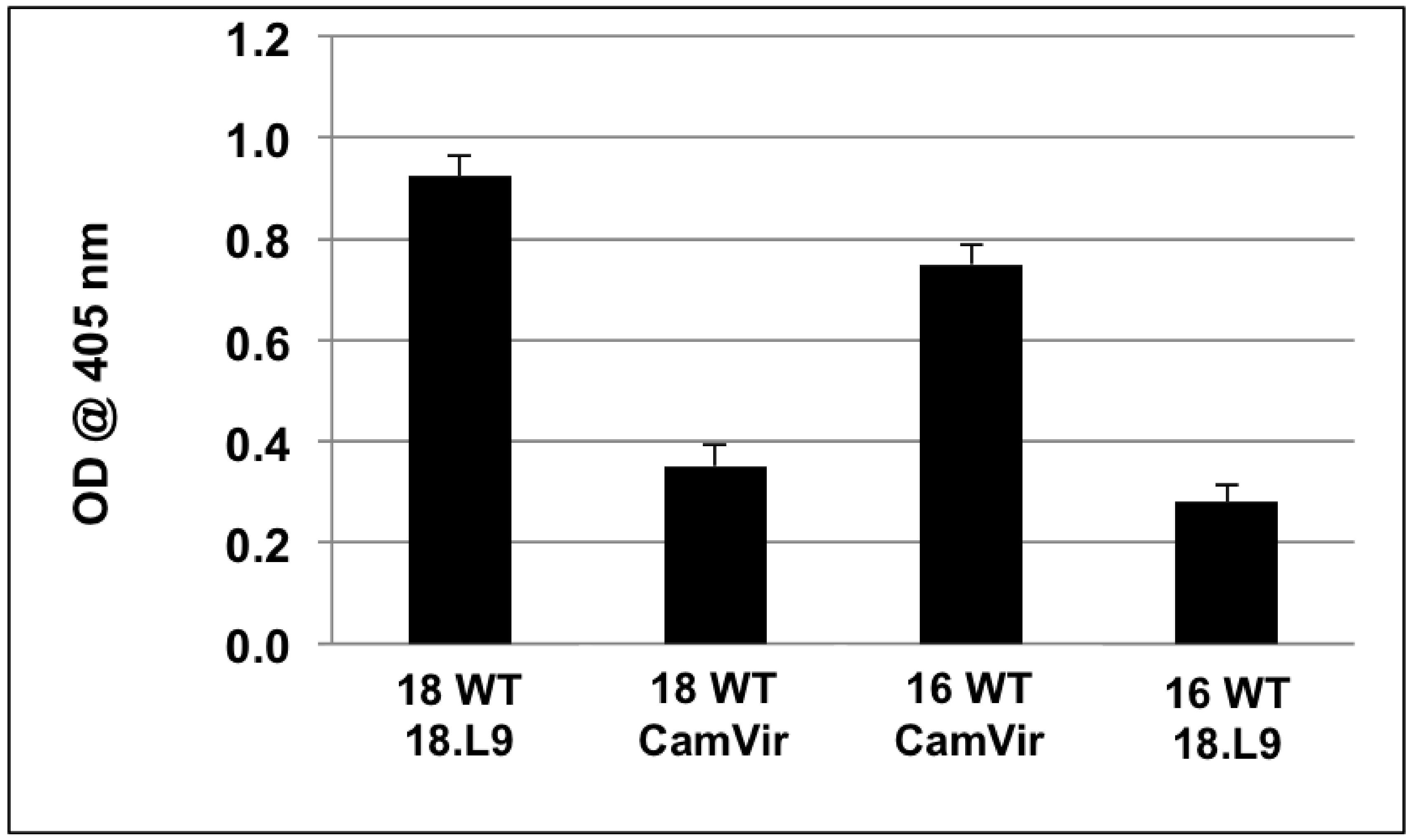

S6. Linear ELISA detects type specific L1 proteins. As a positive control for assay specificity, HPV16 and HPV18 virions were denatured and incubated with the L1-specific antibodies Camvir (HPV16 L1) and 18.L9 (HPV18 L1), respectively, to confirm specific antibody recognition.
